# Genetic Variability and Prediction of T Epitopes of the HPV16 E2 Gene in Asymptomatic Women from Cajamarca, Peru

**DOI:** 10.3390/v17111420

**Published:** 2025-10-25

**Authors:** Eliezer Bonifacio-Velez de Villa, Deysi Aguilar-Luis, Dayana Denegri-Hinostroza, Miguel Angel Aguilar-Luis, Wilmer Silva-Caso, Yordi Tarazona-Castro, Lorena Becerra-Goicochea, Ronald Aquino-Ortega, Angela Cornejo-Tapia, Juana del Valle-Mendoza

**Affiliations:** 1 Biomedicine Laboratory, Research Center of Faculty of Health Sciences, Universidad Peruana de Ciencias Aplicadas, Lima 15023, Perumiguel.aguilar@upc.pe (M.A.A.-L.);; 2 Oncology Unit, Hospital Regional Docente de Cajamarca, Cajamarca 06003, Peru; 3 Professional Academic School of Obstetrics, Universidad Nacional de Cajamarca, Cajamarca 06003, Peru

**Keywords:** human papillomavirus 16, E2 protein, human papillomavirus, epitopes, T-lymphocyte

## Abstract

Background: The HPV16 E2 gene plays a crucial role in viral replication and oncogene regulation. This study aimed to assess the genetic variability of the E2 gene and to identify immunogenic epitopes of the E2 protein. Methods: The E2 gene was amplified and sequenced. T-cell epitope prediction and evaluation were performed using IEDB, NetMHCpan v4.0, NetMHCIIpan v4.1, VaxiJen, ToxNet, and pLM4Alg. Results: Phylogenetic analysis of 47 E2 sequences demonstrated co-circulation of the D (*n* = 4) and A (*n* = 43) HPV16 lineages in Cajamarca. Twenty-eight Single Nucleotide Polymorphism (SNPs) were identified in E2, 21 of which were nonsynonymous. Seventeen variations were associated with positive Papanicolaou (Pap) test results. Epitope prediction identified 2 MHC class I and 27 MHC class II epitopes classified as potentially antigenic, non-toxic, and non-allergenic, with an overall global population coverage across both MHC classes of 99.78%. Conclusions: The A HPV16 lineage predominated among the women studied. The identified SNPs indicate substantial variability in the E2 gene and a relationship with endocervical lesions. In total, 29 E2-derived T-cell epitopes with immunogenic potential were identified.

## 1. Introduction

Human papillomavirus (HPV) is a global health concern because of its association with the development of endocervical lesions and cervical cancer (CC). It is estimated that HPV is linked to more than 600,000 new cases of CC each year, and, unfortunately, more than half of those affected die from this malignancy [1]. In Peru, the prevalence of HPV infection is alarmingly high, affecting a substantial proportion of the female population. In 2020, 4270 cases of CC were reported, leading to 2288 deaths, making it the second leading cause of mortality among women in the country [2,3,4].

To date, more than 200 HPV genotypes have been identified, with high-risk types closely associated with endocervical lesions and CC. Among high-risk HPVs, genotype 16 (HPV16) is highly oncogenic, and its persistent infection is strongly associated with the onset of endocervical lesions and the development of cervical cancer [5]. Notably, this genotype is among the most prevalent worldwide and in Peru [6].

Genomic investigations underscore the importance of studying HPV genetic variability—particularly SNPs—many of which have been associated with increased risk of endocervical lesions and CC [7]. Several of these variants map to the E2 gene, which encodes the HPV E2 protein. E2 plays a key role in viral replication and in regulating transcription of the oncogenes E6 and E7 [8]. Given its expression at multiple stages of infection and critical functional (enzymatic) properties for the viral cycle, E2 stands out as a promising target for therapeutic vaccine development.

Computational epitope prediction—short amino-acid sequences within viral proteins—has become a rational and valuable approach to initiate the design of new therapeutic vaccines capable of stimulating effective immune responses, with encouraging results [9,10].

In this context, the present study aims to predict T-cell epitopes in the HPV16 E2 protein while considering genetic variation within the E2 region observed in our local setting, and to explore their association with endocervical lesions in Peruvian women. This approach will provide valuable insights into HPV biology and enable the proposal of in silico vaccine candidates.

## 2. Materials and Methods

### 2.1. Cervical Sample Collection

A total of 1433 endocervical samples were collected from asymptomatic women who voluntarily attended outpatient gynecological checkups at the Hospital Regional Docente de Cajamarca during 2021–2024, within the framework of regional epidemiological surveillance. Samples were obtained by endocervical swabbing following standard procedures and preserved in a PBS-based transport medium. Clinical–epidemiological data were abstracted from each patient’s medical record. All participants provided written informed consent prior to enrollment.

### 2.2. DNA Extraction and HPV Detection by PCR

Genetic material was extracted from 200 μL of each specimen using the Magbead Viral DNA/RNA Kit (CWBIO, Taizhou, China) according to the manufacturer’s instructions. Detection and genotyping of HPV16 were performed by conventional PCR using the extracted DNA as template. DNA amplification employed Taq DNA polymerase (Kappa Biosystem, Wilmington, MA, USA), specific primers, and cycling conditions previously described in the literature [11].

### 2.3. Amplification and Sequencing of the HPV16 E2 Gene

The HPV16 E2 gene was amplified from DNA of samples positive for genotype 16. Conventional PCR was performed using Taq DNA polymerase (Kappa Biosystem, Wilmington, MA, USA) with primers flanking the E2 gene (1185 bp): HPV16-E2F 5′-CAGATTAAGTTTGCACGAGGACGAG-3′ and HPV16-E2R 5′-CAAAGCAAAAAGCACGCCAGTAA-3′. PCR conditions were: 95 °C for 5 min; 55 cycles of 95 °C for 30 s, 57 °C for 45 s, and 72 °C for 1 min 30 s; followed by 72 °C for 10 min, then hold at 10 °C. Amplicons were resolved by 2% agarose gel electrophoresis (Agarose Type I, Calbiochem, Merck, Darmstadt, Germany) and stained with SYBR™ Safe DNA Gel Stain at a final concentration of 5 μg/mL. Positive PCR products were purified using the SpinPrep™ Gel DNA Kit (Merck) and stored at −80 °C for downstream analyses.

All purified PCR amplicons were sequenced using Oxford Nanopore Technology with the Ligation Sequencing Kit v14 (SQK-LSK114, Oxford Nanopore Technologies, Oxford, UK), following the manufacturer’s instructions. The DNA input was normalized to 200 fmol prior to library preparation. Sequencing was carried out on a MinION device equipped with a Flow Cell (R10.4.1). The resulting reads were processed in Epi2me using the Amplicon workflow (wf-amplicons v1.1.4) to generate consensus sequences.

### 2.4. Identification of Variants in HPV16 E2 and Phylogenetic Analysis

Sequences were aligned to the HPV16 reference (GenBank: NC_001526.3) using ClustalW within MEGA v12.0. Phylogenetic trees for the E2 region were constructed by the neighbor-joining method in MEGA 12.0. Tree reliability was assessed by 1000-replicate bootstrap analysis. Study sequences were compared against representative HPV16 lineage variants retrieved from GenBank (28 sequences): HQ644283.1 (A1), HQ644268.1 (A1), HQ644280.1 (A1), HQ644282.1 (A1), AF536179.1 (A2), HQ644236.1 (A3), HQ644248.1 (A4), HQ644251.1 (A4), AF534061.1 (A4), HQ644235.1 (A4), HQ644240.1 (B1), HQ644290.1 (B1), HQ644238.1 (B1), HQ644298.1 (B3), HQ644237.1 (C), HQ644239.1 (C), HQ644249.1 (C), HQ644250.1 (C), AF472509.1 (C), HQ644257.1 (D1), HQ644279.1 (D2), HQ644281.1 (D2), HQ644263.1 (D2), HQ644277.1 (D2), HQ644247.1 (D3), HQ644253.1 (D3), HQ644255.1 (D3), AF402678.1 (D3).

The E2 protein was modeled to visualize amino acid substitutions derived from SNP analysis. Each of the three functional domains (transactivation, hinge, and DNA-binding) was independently modeled using the AlphaFold Server, employing a template-guided approach. The structural quality of the models was assessed through Ramachandran plot analysis, and the domains were assembled into a complete structure using UCSF Chimera version 1.19.0.

### 2.5. Statistical Analysis

Frequencies of HPV16 E2 variants were computed by automated counting in Python. Associations between E2 variants and a positive Papanicolaou (Pap) test were assessed with Fisher’s exact test. Statistical analyses were performed using SciPy v1.16.0 in Python v3.12.7, with *p* < 0.05 considered statistically significant.

### 2.6. T-Cell Epitope Prediction and Selection

Epitope prediction was conducted from the consensus E2 sequence derived from the phylogenetic analyses using the IEDB server. Cytotoxic T-lymphocyte (CTL) epitopes were predicted with NetMHCpan v4.0, assuming lengths of 8–11 amino acids, restricted to MHC class I supertypes selected by IEDB and those most frequent in South America [9,12]. Helper T-cell epitopes were predicted with NetMHCIIpan v4.1, assuming lengths of 11–18 amino acids, targeted to HLA-DR, HLA-DQ, and HLA-DP alleles selected by IEDB and the most prevalent in South America [9,12].

Predicted epitopes were evaluated for sequence conservation against a panel of 520 E2 sequences retrieved from UniProtKB to confirm their presence across known E2 proteins. Subsequently, antigenicity, toxicity, and allergenicity were assessed using VaxiJen [13], ToxinPred [14], and pLM4Alg [15], respectively. Finally, epitopes meeting criteria for immunogenic potential, non-toxicity, and non-allergenicity were evaluated for population coverage using the IEDB Population Coverage tool.

## 3. Results

### 3.1. Epidemiological Characteristics of the Analyzed Samples

**Figure 1:**
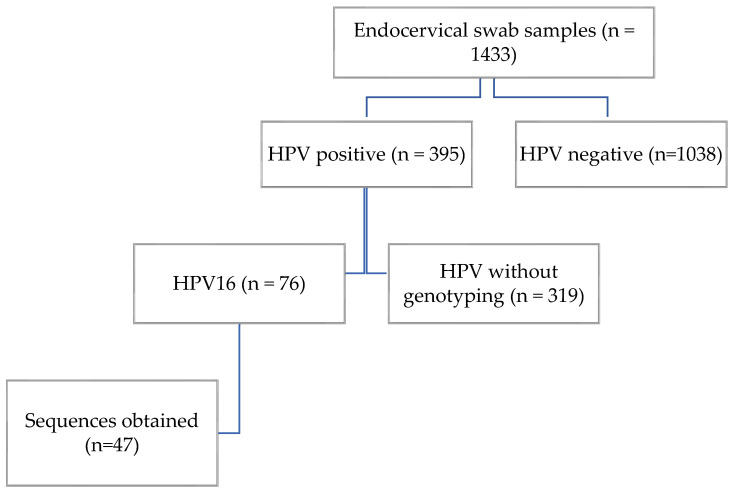
Participant flow diagram.

We analyzed 1433 endocervical swab samples from asymptomatic women by PCR to detect human papillomavirus (HPV). Of these, 395 tested positives for HPV, yielding a prevalence of 27.56%. HPV16 was identified in 76 samples, corresponding to a prevalence of 5.3% in the study population (Figure 1).

### 3.2. Analysis of Variation in the HPV16 E2 Gene

**Figure 2:**
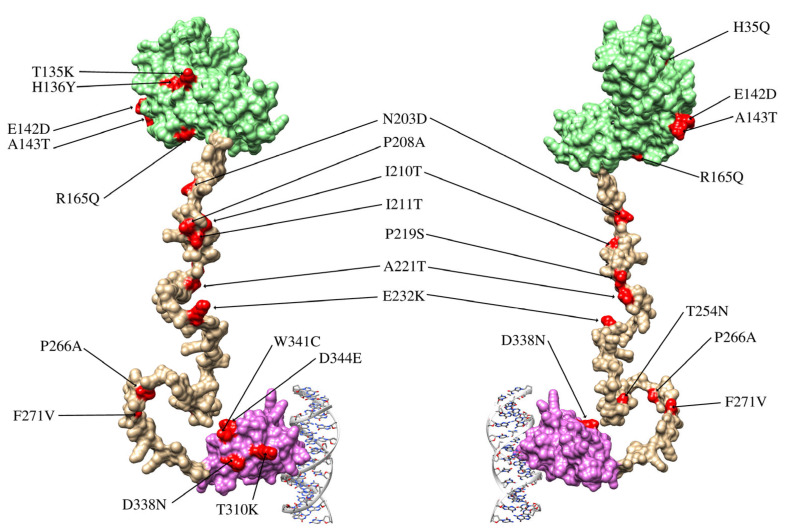
Structure of the E2 protein and locations of the variations identified in this study. The E2 protein comprises three domains: the transactivation domain (light green), the hinge domain (pale gold), and the DNA-binding domain (lilac). The hinge domain lacks a well-defined three-dimensional structure, as its main function is to connect the functional domains of the protein. The figure shows the front and back views of the E2 protein in complex with a DNA fragment.

Out of 76 HPV16-positive samples, only 47 were successfully amplified and sequenced. The remaining samples showed low DNA quality or insufficient amplification of the E2 gene region, possibly due to degradation or low viral load in the clinical specimens. Across these 47 E2 sequences, we identified 28 distinct single nucleotide polymorphisms (SNPs), distributed across the transactivation domain (8 SNPs), the hinge region (12 SNPs), and the DNA-binding domain (8 SNPs). Most SNPs were in the hinge region of the E2 protein, with fewer in the DNA-binding and transactivation domains (Figure 2 and Table 1). The most frequent variation was C3410T, present in 45 samples (95.7%) which is present in lineages A and D. The next most frequent was C3684A, detected in 5 samples (10.6%) which is present in lineage D. Several variations showed a frequency of 8.5%, including C2860A, C3159A, G3182A, T3224A, G3249A, A3362G, C3377G, G3449A, C3516A, T3517C, A3538C, T3566G, T3694A, T3706C, G3778T, C3787A, and T3805G. Variations C3161T and G3416A were observed in 3 samples each (6.4%). Finally, T3118C, A3181C, T3384C, T3387C, C3551G, T3664C, and G3767A each occurred in a single sample (2.1% of sequences).

### 3.3. Phylogenetic Analysis of the HPV16 E2 Gene

**Figure 3:**
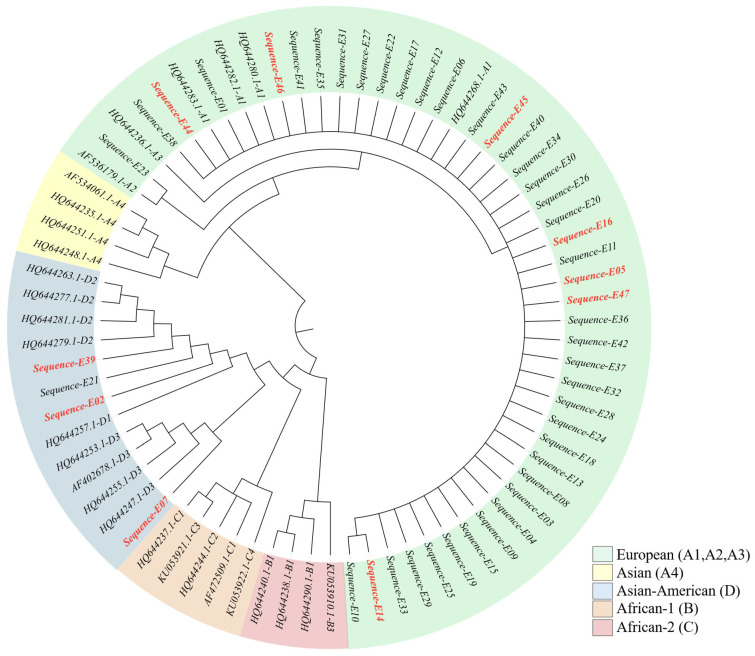
Phylogenetic tree based on the HPV16 E2 gene sequence. Sequences corresponding to patients with a positive Pap smear are shown in red.

Phylogenetic analysis of the 47 nucleotide sequences showed that four clustered within lineage D (Asian American), three of which were derived from Papanicolaou (Pap) test-positive samples. The remaining 43 sequences clustered within lineage A (European). No samples from lineages B (African-1), C (African-2), or A4 (Asian) were detected. These findings indicate a predominance of the A lineage in the study population (Figure 3).

### 3.4. Association Between Variations and Endocervical Lesions

Associations between the presence of each variation and Pap test result were assessed using Fisher’s exact test for small samples. Seventeen variations showed statistically significant associations (Table 1). Specifically, C2860A, C3159A, G3182A, T3224A, G3249A, A3362G, C3377G, G3449A, C3516A, T3517C, A3538C, T3566G, T3694A, T3706C, G3778T, C3787A, and T3805G yielded *p* < 0.05, suggesting a possible association with a positive Pap test result. In contrast, T3118C, T3384C, C3551G, G3767A, C3161T, A3181C, T3387C, C3410T, G3416A, T3664C, and C3684A showed no significant association (*p* ≥ 0.05). Finally, Fisher’s exact test applied to lineage versus Pap result revealed a significant association between the D lineage and a positive Pap test (Table 2).

### 3.5. T-Cell Epitope Prediction: Characteristics and Population Coverage

CD4^+^ T-cell epitope prediction was performed with the IEDB platform using NetMHCIIpan 4.1 EL, evaluating peptide processing and binding against a panel of 29 MHC class II alleles representative of global and South American genetic diversity.

**Figure 4:**
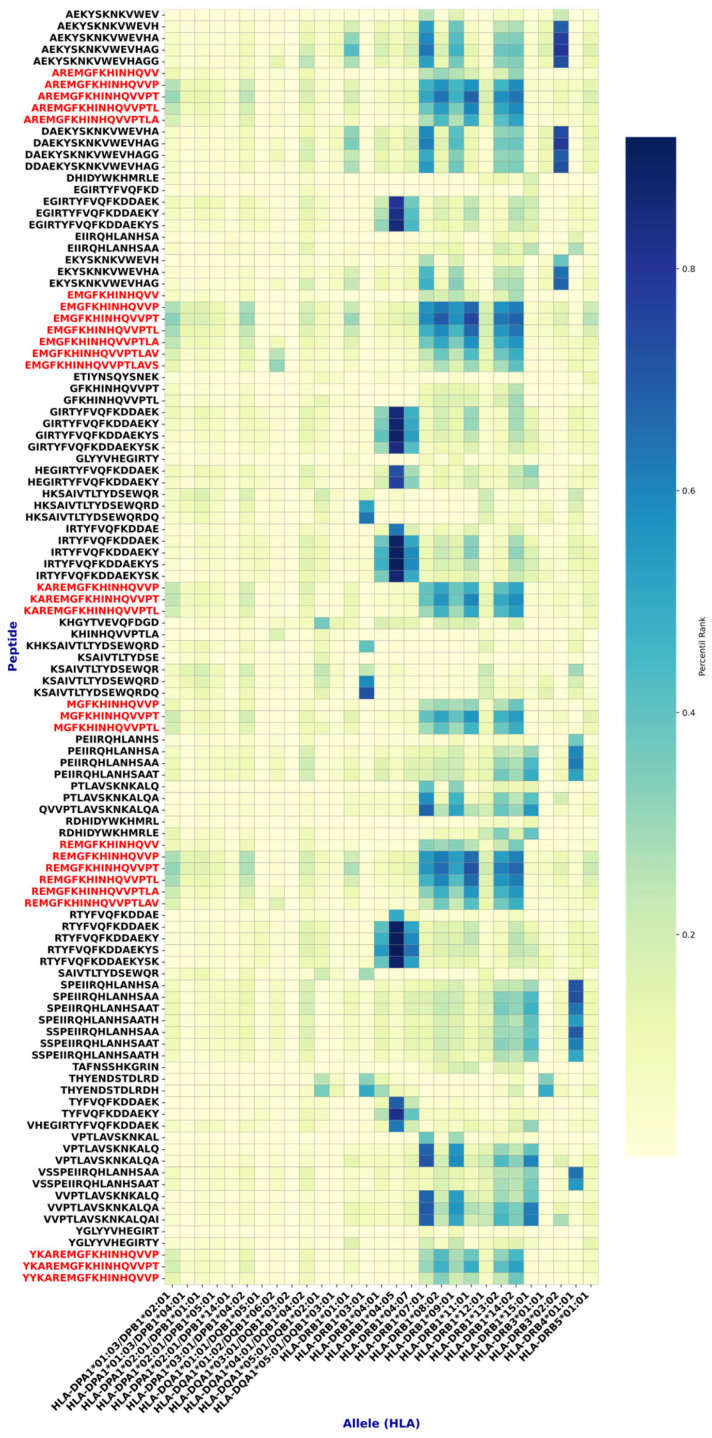
Epitopes found with high affinity for MHC class II. Highly conserved epitopes with a good safety profile are shown in red. Note that these epitopes have a common core: FKHINHQVV.

From all peptides analyzed, 108 were selected for showing binding to ≥25% of the tested MHC-II alleles (Figure 4).

For CD8^+^ T-cell epitopes, NetMHCpan 4.1 EL was applied against a panel of 33 MHC class I alleles. Nine peptides were selected for binding to >25% of the tested alleles (Figure 4).

**Figure 5:**
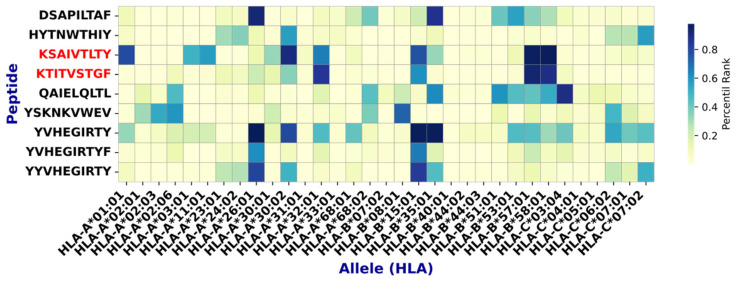
Epitopes found with high affinity for MHC class I. Epitopes with high conservation and good safety profiles are shown in red.

Subsequent analyses identified multiple epitopes with >70% sequence conservation, meeting the criterion to be considered conserved. Among them, 2 MHC-I and 27 MHC-II epitopes (Figure 4 and Figure 5, and Table 3) demonstrated, by in silico analyses, a favorable safety profile (non-toxic and non-allergenic) and immunogenic potential. Notably, the MHC class II epitopes shared a common core motif (FKHINHQVV) across these sequences (Figure 4).

**Figure 6:**
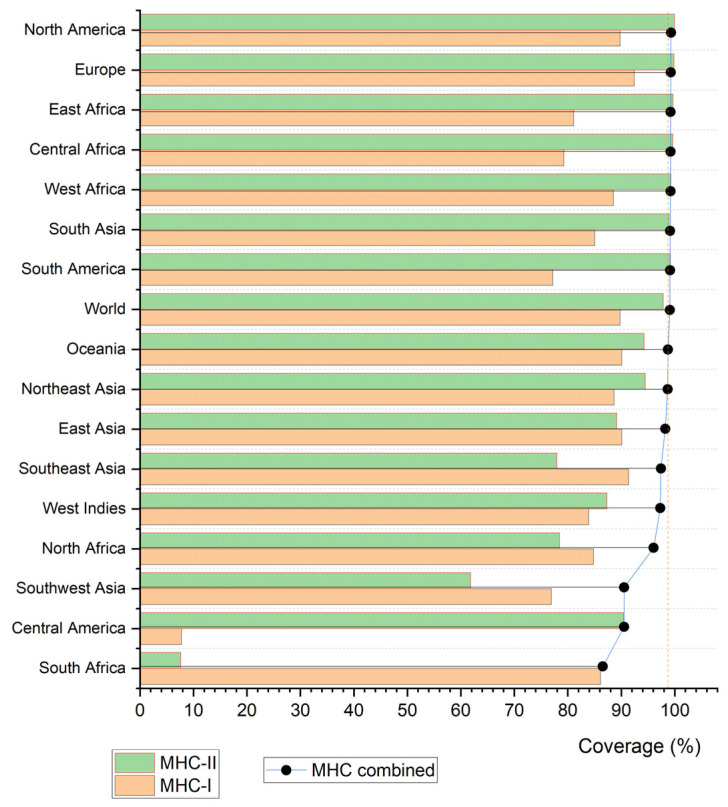
Bar graph illustrating the population coverage of the evaluated epitopes.

Overall, the selected epitopes showed broad combined global population coverage, averaging 99.78%, indicating strong potential to elicit immune responses across diverse populations. Regionally, high coverages were observed in Europe (99.99%), North America (100.00%), and South Asia (99.85%). In contrast, Central America (91.17%) and Southwest Africa (91.20%) exhibited lower, though still substantial, combined coverage—reflecting differences in HLA allele distributions across populations. By class, MHC-I epitopes achieved a mean global coverage of 89.80%, with notable values in Europe (92.44%) and East Asia (90.11%); MHC-II epitopes reached a mean of 97.87%, exceeding 99% in East Africa, South America, and Europe (Figure 6).

## 4. Discussion

HPV prevalence varies globally—from 6.6% in Europe to 22.9% in Africa—and also regionally (Asia 8.3%, North America 13.8%, South America 14.3%, Central America 20.5%) [22], suggesting the influence of geographic, socioeconomic, and cultural factors. Genotype distribution is likewise heterogeneous, with HPV16 among the most prevalent high-risk types. In Peru, overall HPV and HPV16 prevalences are variable: Iwasaki et al. (2014) reported 34.49% (any HPV) and 10.77% (HPV16) [23], whereas del Valle-Mendoza et al. (2021) found 19.08% (any HPV) and 3.24% (HPV16) in Cajamarca [6]. In the present study, we observed an HPV prevalence of 27.56% and an HPV16 prevalence of 5.3% in clinically healthy women—values consistent with del Valle-Mendoza but lower than those of Iwasaki—potentially indicating a lower prevalence of HPV, particularly HPV16, in Cajamarca.

HPV16 is classified into several lineages and sublineages based on genetic variation. Four major lineages are currently recognized, each subdivided into sublineages [24]. Lineage A includes sublineages A1, A2, A3 (European), and A4 (Asian). Lineage B comprises B1 (African-1, Afr1a) and B2 (African-1, Afr1b). Lineage C corresponds to African-2 (Afr2a). Lineage D includes D1 (North American, NA1), D2 (Asian-American, AA2), and D3 (Asian-American, AA1) [25,26]. Several studies have shown that lineage D, particularly the Asian-American (AA) variants, display higher oncogenic potential compared with European variants, possibly due to mutations in viral regulatory genes such as E2 that can alter viral transcription and replication control [16,27,28,29].

Earlier studies (2004, 2007) by Tornesello et al. and Sichero et al. reported that non-European HPV16 variants—such as AA and AFR1—confer a higher risk of cervical intraepithelial lesions than European (EUR) variants [17,27]. Similarly, Schiffman et al. (2010) noted that AFR1, AFR2, and AA variants were more prevalent than EUR and As variants and were associated with greater viral persistence and carcinogenic potential [28]. More recent work by Dai (2018) and Wang (2021) found that Asian and Asian-American HPV16 variants were associated with cervical cancer in Chinese cohorts, suggesting that non-European variants may carry a higher risk of progression to lesions and cancer [16,29]. In our study, most amino acid variations significantly associated with positive Pap results were characteristic of lineage D. This suggests that the statistical significance observed for positive Pap results mainly reflects the contribution of lineage D variants. Given that E2 regulates the transcription of the E6 and E7 oncogenes, alterations found within lineage D may affect this regulation, potentially enhancing viral persistence and the carcinogenic process [16,28,29].

We identified 17 variations significantly associated with endocervical lesions by Fisher’s exact test, in line with previous findings. However, five variations (A3181C, T3384C, T3387C, T3664C, and C3684A) did not show significant associations, contrary to other reports [16,29,30]. Regarding their distribution, we observed greater diversity in the hinge (bridge) domain of E2, consistent with prior studies and suggesting greater tolerance for genetic variation due to the lack of enzymatic functions. The C3410T variation stood out for its high frequency (95.7%), suggesting it is an established variation in circulating HPV16 within the analyzed population. The higher variability observed within the hinge domain may indicate adaptive flexibility, whereas lineage D–specific substitutions in functional regions of E2 could compromise its regulatory role, leading to enhanced oncogene expression and an increased risk of cervical lesion development.

Functionally, the HPV16 E2 protein is pivotal for viral genome replication and repression of the oncogenes E6 and E7, which are implicated in carcinogenesis. Variations in E2 may alter its function and influence viral behavior. In the DNA-binding domain, variations such as G3778T (W341C), T3694A (T313T), and C3684A (T310K) could enhance conformational stability, potentially affecting E2′s repressive capacity on E6/E7 [25,29,31,32]. In the transactivation domain, C2860A (H35Q) may alter E2–E1 interactions and thereby impact viral replication, although its effect remains unclear [31]. Other variations such as T135K and H136Y have been linked to increased HPV16 persistence, although the underlying mechanisms are not fully understood [32]. As for the hinge region, its role in the progression of endocervical lesions has not been extensively studied in HPV16, but in other types (HPV11 and HPV2) it is relevant for nuclear localization, DNA binding, and transactivation activity [33,34], suggesting that variations in this region could modulate E2 function.

T-cell epitope-based vaccines have shown promise in treating viral infections and cancers. For HPV, a vaccine aimed at inducing a robust T-cell response against E2 could complement current L1-based prophylactic vaccines—particularly for individuals already infected or with precancerous lesions [35,36]. Selecting peptides with diverse MHC-binding specificities maximizes population coverage by accounting for MHC polymorphism and allele frequency differences across populations, thereby avoiding ethnic bias. This strategy ensures that epitopes can be presented by a broad array of MHC alleles, which is critical for developing widely applicable vaccines. Accordingly, we identified 2 promising MHC-I epitopes and 27 MHC-II epitopes, the latter exhibiting high similarity to one another. The epitopes also showed a high degree of conservation, supporting their functional relevance and potential for universal HPV vaccine design [37].

We predicted peptides of 8–11 amino acids for MHC-I and 11–18 amino acids for MHC-II, recognizing that MHC-I typically binds shorter epitopes (8–11 aa), whereas MHC-II accommodates a broader length range (~13–25 aa). Notably, in MHC-II, only a central core of ~9 amino acids directly interact with the binding groove [38]. This length flexibility allows identification of a wider variety of candidate epitopes—particularly important for pathogens with high genetic variability such as HPV.

The epitopes identified here display a high degree of conservation, underscoring their likely functional importance in immune recognition. This is especially relevant given that HPV16 variants can harbor amino-acid substitutions that may alter adaptive immune responses. For example, variations W341C and D344E in the EUR variant lie within the epitope HKSAIVTLTYDSEWQRDQ [39]. Likewise, E142D and A143T, detected in this study, map to regions corresponding to epitopes EEASVTVVEGQVDYY and YICEEASVTV [39,40], suggesting a potential impact on antigen presentation. Similarly, R145Q may affect the structure or function of the YVHEGIRTY epitope, diminishing immune recognition [41]. In future work, we propose investigating the functional impact of the variations identified within known epitopes to validate their effects on antigen presentation and immune responses.

Taken together, the observed variations in HPV16 E2 may favor immune-evasion mechanisms, facilitating viral persistence in the host. These findings highlight the need to incorporate HPV16 genetic diversity into therapeutic and vaccine strategies to achieve effective immune responses against diverse viral variants.

## 5. Conclusions

We identified an HPV16 prevalence of 5.3% among asymptomatic women in the Cajamarca region. Analysis of the E2 gene revealed substantial genetic diversity, with 28 single-nucleotide polymorphisms (SNPs) distributed across the gene, 17 of which were significantly associated with positive Papanicolaou (Pap) test results. In silico epitope prediction yielded 29 candidates with high predicted MHC affinity that were immunogenic, non-toxic, non-allergenic, and highly conserved across the HPV16 variants analyzed. These epitopes demonstrated broad population coverage, underscoring their potential utility for developing more inclusive and effective vaccine strategies. Taken together, our findings highlight the importance of incorporating HPV16 genetic variability into the design of future diagnostic and prevention tools.

## Figures and Tables

**Table 1:** Variations identified in HPV16 (European sublineage A1, GenBank: NC_001526.3) and their relationship with Pap smear results. *p* values obtained from Fisher’s exact test (FET) < 0.05, indicating statistical significance, are shown in bold.

Variation	FET (*p*-Value)	Frecuency (%)	Number of Variant Samples	Substitution	Domain in the E2 Protein	Associated with CCUb (*p* < 0.05)	Reference
C2860A	**0.026**	8.5	4	H35Q	Transactivation Domain	Yes	[16]
T3118C	1.000	2.1	1	-		-	
C3159A	**0.026**	8.5	4	T135K		Yes	[17,18]
C3161T	0.110	6.4	3	H136Y		-	[19]
A3181C	0.213	2.1	1	E142D		Yes	[16]
G3182A	**0.026**	8.5	4	A143T		Yes	[16]
T3224A	**0.026**	8.5	4	L157I		Yes	[16]
G3249A	**0.026**	8.5	4	R165Q		Yes	[17,18]
A3362G	**0.026**	8.5	4	N203D	Hinge Region	Yes	[16]
C3377G	**0.026**	8.5	4	P208A		Yes	[16]
T3384C	1.000	2.1	1	I210T		Yes	[17,18]
T3387C	0.213	2.1	1	I211T		Yes	[16,17]
C3410T	0.384	95.7	45	P219S		No	[17,18]
G3416A	0.110	6.4	3	A221T		No	[20]
G3449A	**0.026**	8.5	4	E232K		Yes	[17,18]
C3516A	**0.026**	8.5	4	T254N		Yes	[16]
T3517C	**0.026**	8.5	4	-		Yes	[16]
A3538C	**0.026**	8.5	4	-		Yes	[16]
C3551G	1.000	2.1	1	P266A		-	
T3566G	**0.026**	8.5	4	F271V		Yes	[16]
T3664C	0.213	2.1	1	-	DNA Binding	Yes	[16]
C3684A	0.057	10.6	5	T310K		Yes	[17,18]
T3694A	**0.026**	8.5	4	-		Yes	[16]
T3706C	**0.026**	8.5	4	-		Yes	[16]
G3767A	1.000	2.1	1	D338N		No	[21]
G3778T	**0.026**	8.5	4	W341C		Yes	[16]
C3787A	**0.026**	8.5	4	D344E		Yes	[17,18]
T3805G	**0.026**	8.5	4	-		Yes	[16]

**Table 2:** Association between HPV16 lineages and Pap smear results (Fisher’s exact test, *p* = 0.026).

HPV16 Lineage	Pap-Positive (*n* = 10)	Pap-Negative (*n* = 37)
A (European)	7	36
D (Asian-American)	3	1

**Table 3:** Characterization of epitopes with high immunological potential.

Epitope	Alelos/Class	Matches Identity 100%	Minimum Identity	Ubicación en la Proteina	MW	pI	Charge	Instability Index	Aliphatic Index	GRAVY
KSAIVTLTY	15/MHC-I	97.32% (508/522)	88.89%	TD	995.17	8.59	0.55	−9.98	0.11	0.77
KTITVSTGF	9/MHC-I	98.47% (514/522)	88.89%	TD	953.09	8.75	0.55	−0.54	0.11	0.48
EMGFKHINHQVVP	21/MHC-II	95.21% (497/522)	0.8462	BD	1535.77	7.01	−0.26	40.48	0.08	−0.4
MGFKHINHQVVP	21/MHC-II	95.59% (499/522)	0.9167	BD	1406.65	8.54	0.36	43.02	0.08	−0.14
EMGFKHINHQVVPT	19/MHC-II	95.02% (496/522)	0.8571	BD	1636.87	7.01	−0.26	38.31	0.07	−0.42
EMGFKHINHQVV	18/MHC-II	95.21% (497/522)	0.8333	BD	1438.65	7.01	−0.26	26.97	0.08	−0.3
REMGFKHINHQVVP	18/MHC-II	95.21% (497/522)	0.8571	BD	1691.95	8.76	0.63	38.31	0.07	−0.69
MGFKHINHQVVPT	18/MHC-II	95.40% (498/522)	0.9231	BD	1507.76	8.54	0.36	40.48	0.08	−0.18
REMGFKHINHQVVPT	18/MHC-II	95.02% (496/522)	0.8667	BD	1793.06	8.76	0.63	36.42	0.07	−0.69
AREMGFKHINHQVVPT	16/MHC-II	94.83% (495/522)	0.875	BD	1864.14	8.8	0.68	34.77	0.06	−0.54
EMGFKHINHQVVPTL	16/MHC-II	94.83% (495/522)	0.8667	BD	1750.03	7.01	−0.26	36.42	0.07	−0.14
REMGFKHINHQVVPTL	16/MHC-II	94.83% (495/522)	0.875	BD	1906.22	8.76	0.63	34.77	0.06	−0.41
AREMGFKHINHQVVP	15/MHC-II	95.02% (496/522)	0.8667	BD	1763.03	8.8	0.68	36.42	0.07	−0.53
KAREMGFKHINHQVVPT	15/MHC-II	94.83% (495/522)	0.8824	BD	1992.31	9.99	1.63	33.31	0.06	−0.74
AREMGFKHINHQVVPTL	14/MHC-II	94.64% (494/522)	0.8824	BD	1977.29	8.8	0.68	33.31	0.06	−0.28
EMGFKHINHQVVPTLA	15/MHC-II	94.64% (494/522)	0.875	BD	1821.11	7.01	−0.26	34.77	0.06	−0.02
REMGFKHINHQVV	15/MHC-II	95.21% (497/522)	0.8462	BD	1594.84	8.76	0.63	25.67	0.08	−0.62
KAREMGFKHINHQVVPTL	14/MHC-II	94.64% (494/522)	0.8889	BD	2105.47	9.99	1.63	32.02	0.06	−0.48
YKAREMGFKHINHQVVPT	14/MHC-II	94.83% (495/522)	0.8889	BD	2155.48	9.7	1.62	32.02	0.11	−0.77
REMGFKHINHQVVPTLA	14/MHC-II	94.64% (494/522)	0.8824	BD	1977.29	8.76	0.63	33.31	0.06	−0.28
AREMGFKHINHQVVPTLA	14/MHC-II	94.44% (493/522)	0.8889	BD	2048.37	8.8	0.68	32.02	0.06	−0.17
MGFKHINHQVVPTL	14/MHC-II	95.21% (497/522)	0.9286	BD	1620.92	8.54	0.36	38.31	0.07	0.1
KAREMGFKHINHQVVP	13/MHC-II	95.02% (496/522)	0.875	BD	1891.2	9.99	1.63	34.77	0.06	−0.74
REMGFKHINHQVVPTLAV	15/MHC-II	93.49% (488/522)	0.8889	BD	2076.42	8.76	0.63	32.02	0.06	−0.03
EMGFKHINHQVVPTLAV	14/MHC-II	93.49% (488/522)	0.8824	BD	1920.24	7.01	−0.26	33.31	0.06	0.23
YKAREMGFKHINHQVVP	12/MHC-II	95.02% (496/522)	0.8824	BD	2054.38	9.7	1.62	33.31	0.12	−0.77
EMGFKHINHQVVPTLAVS	11/MHC-II	93.49% (488/522)	0.8889	BD	2007.32	7.01	−0.26	32.02	0.06	0.17
YYKAREMGFKHINHQVVP	11/MHC-II	95.02% (496/522)	0.8889	BD	2217.55	9.53	1.62	38.87	0.17	−0.8
AREMGFKHINHQVV	11/MHC-II	95.02% (496/522)	0.8571	BD	1665.92	8.8	0.68	24.55	0.07	−0.45

## Data Availability

The nucleotide sequences generated in this study have been deposited in the NCBI GenBank database under accession numbers PX439089–PX439135. Dataset presented in the study are openly available in: https://doi.org/10.6084/m9.figshare.30267019 (accessed on 2 October 2025).

## References

[1] Kombe Kombe A.J., Li B., Zahid A., Mengist H.M., Bounda G.-A., Zhou Y., Jin T. (2021). Epidemiology and Burden of Human Papillomavirus and Related Diseases, Molecular Pathogenesis, and Vaccine Evaluation. Front. Public Health.

[2] Silva-Caso W., Olivera-Irazábal M., León-Álvarez P., Del Valle L.J., Díaz-Estacio S., Vargas M., Ruiz J., Bermúdez-García A., del Valle Mendoz J. (2014). Identification of human papillomavirus as a preventive strategy for cervical cancer in asymptomatic women in the Peruvian Andes. Asian Pac. J. Trop. Med..

[3] Sung H., Ferlay J., Siegel R.L., Laversanne M., Soerjomataram I., Jemal A., Bray F. (2021). Global Cancer Statistics 2020: GLOBOCAN Estimates of Incidence and Mortality Worldwide for 36 Cancers in 185 Countries. CA Cancer J. Clin..

[4] de Martel C., Georges D., Bray F., Ferlay J., Clifford G.M. (2020). Global burden of cancer attributable to infections in 2018: A worldwide incidence analysis. Lancet Glob. Health.

[5] Poniewierza P., Panek G. (2022). Cervical Cancer Prophylaxis—State-of-the-Art and Perspectives. Healthcare.

[6] del Valle-Mendoza J., Becerra-Goicochea L., Aguilar-Luis M.A., Pinillos-Vilca L., Carrillo-Ng H., Silva-Caso W., Palomares-Reyes C., Taco-Masias A.-A., Aquino-Ortega R., Tinco-Valdez C. (2021). Genotype-specific prevalence of human papillomavirus infection in asymptomatic Peruvian women: A community-based study. BMC Res. Notes.

[7] Hirose Y., Yamaguchi-Naka M., Onuki M., Tenjimbayashi Y., Tasaka N., Satoh T., Tanaka K., Iwata T., Sekizawa A., Matsumoto K. (2020). High Levels of Within-Host Variations of Human Papillomavirus 16 E1/E2 Genes in Invasive Cervical Cancer. Front. Microbiol..

[8] Anayannis N.V., Schlecht N.F., Ben-Dayan M., Smith R.V., Belbin T.J., Ow T.J., Blakaj D.M., Burk R.D., Leonard S.M., Woodman C.B. (2018). Association of an intact E2 gene with higher HPV viral load, higher viral oncogene expression, and improved clinical outcome in HPV16 positive head and neck squamous cell carcinoma. PLoS ONE.

[9] Requena D., Médico A., Chacón R.D., Ramírez M., Marín-Sánchez O. (2020). Identification of Novel Candidate Epitopes on SARS-CoV-2 Proteins for South America: A Review of HLA Frequencies by Country. Front. Immunol..

[10] Zhu L., Cui X., Yan Z., Tao Y., Shi L., Zhang X., Yao Y., Shi L. (2024). Design and evaluation of a multi-epitope DNA vaccine against HPV16. Hum. Vaccines Immunother..

[11] Carrillo-Ng H., Becerra-Goicochea L., Tarazona-Castro Y., Pinillos-Vilca L., del Valle L.J., Aguilar-Luis M.A., Tinco-Valdez C., Silva-Caso W., Martins-Luna J., Peña-Tuesta I. (2021). Variations in cervico-vaginal microbiota among HPV-positive and HPV-negative asymptomatic women in Peru. BMC Res. Notes.

[12] Dhanda S.K., Mahajan S., Paul S., Yan Z., Kim H., Jespersen M.C., Jurtz V., Andreatta M., Greenbaum J.A., Marcatili P. (2019). IEDB-AR: Immune epitope database—Analysis resource in 2019. Nucleic Acids Res..

[13] Doytchinova I.A., Flower D.R. (2007). VaxiJen: A server for prediction of protective antigens, tumour antigens and subunit vaccines. BMC Bioinform..

[14] Rathore A.S., Arora A., Choudhury S., Tijare P., Raghava G.P.S. (2023). ToxinPred 3.0: An improved method for predicting the toxicity of peptides. Comput. Biol. Med..

[15] Du Z., Xu Y., Liu C., Li Y. (2024). pLM4Alg: Protein Language Model-Based Predictors for Allergenic Proteins and Peptides. J. Agric. Food Chem..

[16] Dai S., Yao Y., Yan Z., Zhou Z., Shi L., Wang X., Sun L., Zhang R., Yao Y. (2018). The association of human papillomavirus type 16 E2 variations with cervical cancer in a Han Chinese population. Infect. Genet. Evol..

[17] Wang L., Wang F., Fu S., Zhang C., Zhe X., Li H., Li D., Shao R., Pan Z. (2021). Analysis of genetic variation in human papillomavirus type 16 E1 and E2 in women with cervical infection in Xinjiang, China. BMC Med. Genom..

[18] Cheung J.L., Cheung T.H., Yu M.Y., Chan P.K. (2013). Virological characteristics of cervical cancers carrying pure episomal form of HPV16 genome. Gynecol. Oncol..

[19] Smith B., Chen Z., Reimers L., van Doorslaer K., Schiffman M., Desalle R., Herrero R., Yu K., Wacholder S., Wang T. (2011). Sequence imputation of HPV16 genomes for genetic association studies. PLoS ONE.

[20] Zu Y., Ou Z., Wu D., Liu W., Liu L., Wu D., Zhao Y., Ren P., Zhang Y., Li W. (2021). Genetic characteristics of human papillomavirus type 16, 18, 52 and 58 in southern China. Genomics.

[21] Brown C., Kowalczyk A.M., Taylor E.R., Morgan I.M., Gaston K. (2008). P53 represses human papillomavirus type 16 DNA replication via the viral E2 protein. Virol. J..

[22] Crow J.M. (2012). HPV: The global burden. Nature.

[23] Iwasaki R., Galvez-Philpott F., Arias-Stella J., Arias-Stella J. (2014). Prevalence of high-risk human papillomavirus by cobas 4800 HPV test in urban Peru. Braz. J. Infect. Dis..

[24] Cornet I., Gheit T., Iannacone M.R., Vignat J., Sylla B.S., Del Mistro A., Franceschi S., Tommasino M., Clifford G.M. (2013). HPV16 genetic variation and the development of cervical cancer worldwide. Br. J. Cancer.

[25] Kahla S., Hammami S., Kochbati L., Chanoufi M.B., Oueslati R. (2018). HPV16 E2 variants correlated with radiotherapy treatment and biological significance in cervical cell carcinoma. Infect. Genet. Evol..

[26] Wu H., Wu E., Ma L., Zhang G., Shi Y., Huang J., Zha X. (2015). Lineage distribution and E2 sequence variation of high-risk human papillomavirus types isolated from patients with cervical cancer in Sichuan province, China. Arch. Virol..

[27] Sichero L., Ferreira S., Trottier H., Duarte-Franco E., Ferenczy A., Franco E.L., Villa L.L. (2007). High grade cervical lesions are caused preferentially by non-European variants of HPVs 16 and 18. Int. J. Cancer.

[28] Tornesello M.L., Duraturo M.L., Salatiello I., Buonaguro L., Losito S., Botti G., Stellato G., Greggi S., Piccoli R., Pilotti S. (2004). Analysis of human papillomavirus type-16 variants in Italian women with cervical intraepithelial neoplasia and cervical cancer. J. Med. Virol..

[29] Schiffman M., Rodriguez A.C., Chen Z., Wacholder S., Herrero R., Hildesheim A., Desalle R., Befano B., Yu K., Safaeian M. (2010). A population-based prospective study of carcinogenic human papillomavirus variant lineages, viral persistence, and cervical neoplasia. Cancer Res..

[30] Schiller J.T., Müller M. (2015). Next generation prophylactic human papillomavirus vaccines. Lancet Oncol..

[31] van der Weele P., Meijer C.J.L.M., King A.J. (2017). Whole-Genome Sequencing and Variant Analysis of Human Papillomavirus 16 Infections. J. Virol..

[32] Zhang L., Liao H., Yang B., Geffre C.P., Zhang A., Zhou A., Cao H., Wang J., Zhang Z., Zheng W. (2015). Variants of human papillomavirus type 16 predispose toward persistent infection. Int. J. Clin. Exp. Pathol..

[33] Gao C., Pan M.-M., Lei Y.-J., Tian L.-Q., Jiang H.-Y., Li X.-L., Shi Q., Tian C., Yuan Y.K., Fan G.X. (2012). A point mutation in the DNA-binding domain of HPV-2 E2 protein increases its DNA-binding capacity and reverses its transcriptional regulatory activity on the viral early promoter. BMC Mol. Biol..

[34] Zou N., Lin B.Y., Duan F., Lee K.-Y., Jin G., Guan R., Yao G., Lefkowitz E.J., Broker T.R., Chow L.T. (2000). The Hinge of the Human Papillomavirus Type 11 E2 Protein Contains Major Determinants for Nuclear Localization and Nuclear Matrix Association. J. Virol..

[35] Melief C.J.M., van der Burg S.H. (2008). Immunotherapy of established (pre)malignant disease by synthetic long peptide vaccines. Nat. Rev. Cancer.

[36] Trimble C.L., Morrow M.P., Kraynyak K.A., Shen X., Dallas M., Yan J., Edwards L., Parker R.L., Denny L., Giffear M. (2015). Safety, efficacy, and immunogenicity of VGX-3100, a therapeutic synthetic DNA vaccine targeting human papillomavirus 16 and 18 E6 and E7 proteins for cervical intraepithelial neoplasia 2/3: A randomised, double-blind, placebo-controlled phase 2b trial. Lancet.

[37] Bui H.-H., Sidney J., Li W., Fusseder N., Sette A. (2007). Development of an epitope conservancy analysis tool to facilitate the design of epitope-based diagnostics and vaccines. BMC Bioinform..

[38] Rizarullah, Aditama R., Giri-Rachman E.A., Hertadi R. (2024). Designing a Novel Multiepitope Vaccine from the Human Papilloma Virus E1 and E2 Proteins for Indonesia with Immunoinformatics and Molecular Dynamics Approaches. ACS Omega.

[39] Kristensen N.P., Dionisio E., Bentzen A.K., Tamhane T., Kemming J.S., Nos G., Voss L.F., Hansen U.K., Lauer G.M., Hadrup S.R. (2024). Simultaneous analysis of pMHC binding and reactivity unveils virus-specific CD8 T cell immunity to a concise epitope set. Sci. Adv..

[40] Stellato G., Paavonen J., Nieminen P., Hibma M., Vilja P., Lehtinen M. (1997). Diagnostic phase antibody response to the human papillomavirus type 16 E2 protein is associated with successful treatment of genital HPV lesions with systemic interferon alpha-2b. Clin. Diagn. Virol..

[41] Bhatt K.H., Neller M.A., Srihari S., Crooks P., Lekieffre L., Aftab B.T., Liu H., Smith C., Kenny L., Porceddu S. (2020). Profiling HPV-16-specific T cell responses reveals broad antigen reactivities in oropharyngeal cancer patients. J. Exp. Med..

